# A Comparative Cytotoxic Evaluation of Disulfiram Encapsulated PLGA Nanoparticles on MCF-7 Cells

**Published:** 2017-04-01

**Authors:** Hamidreza Fasehee, Ardeshir Ghavamzadeh, Kamran Alimoghaddam, Seyed-Hamidollah Ghaffari, Shahab Faghihi

**Affiliations:** 1 Stem Cell and Regenerative Medicine Group, National Institute of Genetic Engineering and Biotechnology (NIGEB), Tehran, Iran; 2 Hematology, Oncology and Stem Cell Transplantation Research Center, Shariati Hospital, Tehran University of Medical Sciences, Tehran, Iran

**Keywords:** Disulfiram, Poly (Lactic-co-glycolic acid), Nanoparticles, MCF-7 cells

## Abstract

**Background:** Disulfiram is oral aldehyde dehydrogenase (ALDH) inhibitor that has been used in the treatment of alcoholism. Recent studies show that this drug has anticancer properties; however, its rapid degradation has limited its clinical application. Encapsulation of disulfiram polymeric nanoparticles (NPs) may improve its anticancer activities and protect rapid degradation of the drug.

**Materials and** **Methods:** A poly (lactide-co-Glycolide) (PLGA) was developed for encapsulation of disulfiram and its delivery into breast cancer cells. Disulfiram encapsulated PLGA NPs were prepared by nanoprecipitation method and were characterized by Scanning Electron Microscopy (SEM). The loading and encapsulation efficiency of NPs were determined using UV-Visible spectroscopy. Cell cytotoxicity of free and encapsulated form of disulfiram is also determined using MTT assay.

**Results:** Disulfiram encapsulated PLGA NPs had uniform size with 165 nm. Drug loading and entrapment efficiency were 5.35 ±0.03% and 58.85±1.01%. The results of MTT assay showed that disulfiram encapsulated PLGA NPs were more potent in induction of apoptosis compare to free disulfiram.

**Conclusion: **Based on the results obtained in the present study it can be concluded that encapsulation of disulfiram with PLGA can protect its degradation in improve its cytotoxicity on breast cancer cells.

## Introduction

 Disulfiram (Antabus) is an inhibitor of aldehyde dehydrogenase (ALDH) that has been used in the treatment of alcoholism since the 1940’s.^1^^-^^6^Recent reports indicated that disulfiram have some anti-cancer properties.^1^^-^^4^^,^^6^^-^^9^ The biological activity of disulfiram against cancer cells could be attributed to the ability of this drug to bind the divalent cations such as copper and zinc.^10^Disulfiram was shown to inhibit the matrix metalloproteinase (MMPs),^10^ proteasome,^5^^,^^8^^,^^11^^,^^12^NF-kB signaling pathway,^13^^-^^16^aldehyde dehydrogenase (ALDH), P-glycoproteins (P-gp)^5^ and superoxide dismutase (SOD).^1^ One important mechanism of disulfiram cytotoxicity is to generate high amount of reactive oxygen species (ROS) inside the cells which leads to mitochondrial permeabilization and apoptosis initiation^1^^,^^17^^,^^18^ Because of its significant anti-cancer effects and its low price, disulfiram is considered as “Repurposing drug” in cancer therapy and many researches have been focused on the application of this drug in hematological malignancies and solid tumors.^19^ However, there is still some barrier to translate this drug from lab to clinic. The currently available oral formulation of disulfiram is unstable in acidic gastric environment and is also rapidly degraded in blood stream with a half-life less than 4 minutes.^14^^,^^16^

So, an efficient drug delivery system is essential to clinically use Disulfiram in cancer treatment. In this study, we developed a new formulation for Disulfiram using PLGA nanoparticles (NPs). PLGA is a FDA approved synthetic polymer which have been used in synthesis of nanoparticles applicable in drug delivery.^20^ PLGA NPs can protect the disulfiram from rapid degradation, prolong their presence in blood stream and give the possibility of sustained release of drug into tumor site. 

## MATERIALSANDMETHODS

**Materials**

Poly (vinyl alcohol) (PVA), Poly (lactide-co-glycolide) (PLGA) (504H) and Dialysis tubing cellulose membrane with MWCO: 12kDa were obtained from Sigma-Aldrich; disulfiram, methanol and dimethyl sulfoxide (DMSO) was obtained from Merck (Germany); Fetal bovine serum (FBS), DMEM media, PBS buffer, Trypsin/EDTA and penicillin-streptomycin were purchased from GIBCO (USA); MTT(3-(4,5-dimethylthiazol-2-yl)-2,5- diphenyltetrazolium bromide) assay kit was obtained from Roche (Germany).

**Nanoparticle Preparation**

**Fig. 1:**
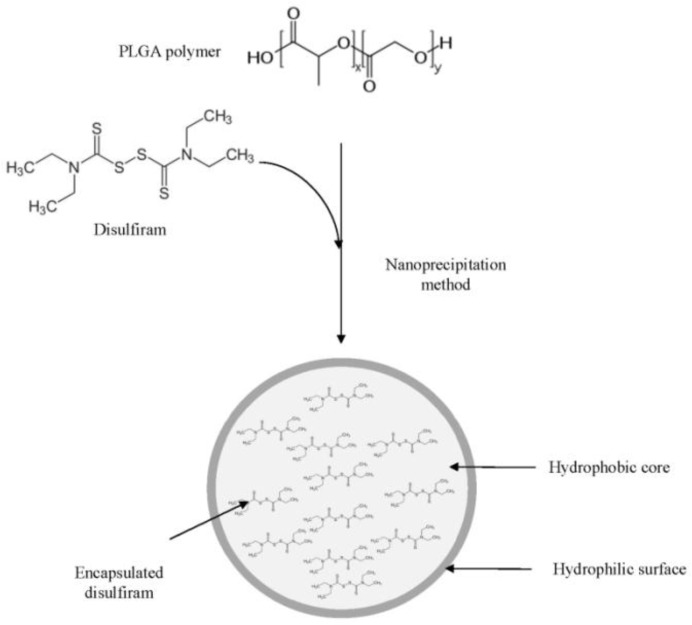
Preparation of disulfiram encapsulated PLGA nanoparticles using nanoprecipitation method

The polymer (PLGA) and disulfiram was dissolved in a DMSO to form the diffusing phase. The ratio of drug (disulfiram) to polymer (PLGA) was 1:10 (w/w). The resultant mixture was then added to the dispersing phase (PVA 0.5% in water) by means of a syringe positioned with the needle directly in the medium under moderate magnetic stirring. The ratio of diffusing phase to dispersing phase was 1:20 (v/v).The freshly formed nanoparticles were then dialyzed (Dialysis tubing cellulose membrane with MWCO: 12kDa) against water for 24 hours. After that, the nanoparticles were centrifuged at 20000g for 15min, washed 4 times with distilled water in order to gradually remove DMSO and free disulfiram. Finally, the nanoparticles were freeze-dried and kept in refrigerator.^   20 ^(Figure 1)

**Characterization of NPs**

The mean particle size of the NPs was determined by dynamic light scatter using photon correlation spectroscopy. The measurements were performed using a Zetasizer Nano ZS (Malvern Instruments Ltd, Malvern, UK) equipped with a helium-neon laser at 25°C and a scattering angle of 173°C.^20^

The morphological examination of NPs was performed using SEM analysis. The drug loading and encapsulation efficiency of disulfiram in NPs were determined as follows: 10 mg of dried NPs was dispersed in 10 mL phosphate-buffered saline (PBS) solution (pH 7.4) to obtain a final NPs concentration (10 mg/mL). About 10 L of the sample was removed from the nanoparticle suspension, and then 90 L of DMSO was added to destruct the NPs. After the sample was vortexed for 30 s, 900 L methanol was added to precipitate insoluble polymers and concentration of disulfiram was analyzed using UV-Visible spectroscopy in 433 nm. The encapsulation efficiency (EE) was determined as the mass ratio of entrapped disulfiram in NPs to the theoretical amount of drug used in their preparation. 

**Stability of Free and Encapsulated Disulfiram**

Free disulfiram (0.01 mg/mL) or encapsulated disulfiram (0.01 mg disulfiram equivalent/mL) was dissolvedin PBS containing 10% FBS and kept at 37˚Cshaking incubator with shaking rate of 100 rpm. In predetermined time-points (0, 24, 48 and 72 h), 0.5 mL of solution was freeze-dried and dissolved in DMSO and the presence of disulfiram in samples wasidentified using spectrophotometry (433 nm). 

**MTT Cell Proliferation Assay**

The cytotoxicity of disulfiram loaded NPs on Breast cancer cell line (MCF7) was determined via the reduction of3-(4, 5-dimethythiazol-2-yl)-2, 5-diphenyl tetrazolium bromide (MTT, Sigma) to (dark purple) Formosan. Briefly, MCF7 cells were seeded at 5000/well in flat-bottom96-well culture and incubated at 37 °C at 5%. CO2 in DMEM medium with various concentrations of NPs and free disulfiram for 24 h. After removing the media, cells were further incubated with MTT solution (5 mg/ml in PBS) at 37 °C for 3 h, and the untreated cells were defined as the control group. Then, supernatant medium was discarded after centrifugation, and DMSO was added to dissolve precipitated Formosan. Finally, absorbance of converted dye was measured in an ELISA reader at wavelength of 570 nm.^20^

**Statistical Analysis**

Each experiment was performed at least in triplicate. All results are summarized as means ± standard deviation and statistical differences were determined by analysis of variance followed by Student’s t-test. Results were considered statistically significant when p<0.05.

## Results

**Drug Loading and Encapsulation Efficiency**

Drug loading and encapsulation efficiency of NPs have been measured by UV visible spectrophotometer. The results are shown in Table 1.The drug loading and encapsulation efficiency of NPs was 5.35 ± 0.03% and 58.85 ± 1.01%, respectively. This data confirms that the NPs are effective for encapsulating the disulfiram and have reasonable encapsulation efficiency.

**Table 1:** Drug loading and Encapsulation efficiency of disulfiram encapsulated PLGA NPs

NPs	Drug loading (%)	Encapsulation efficiency (%)
PLGA	5.35± 0.03	58.85±1.01

**Morphology of NPs**

**Fig. 2:**
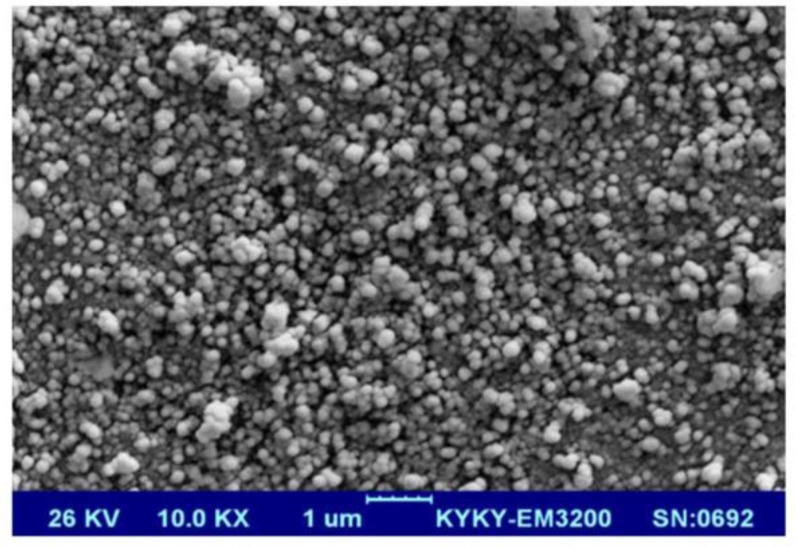
SEM image of disulfiram encapsulated PLGA NPs

The results of Zetasizer analysis showed that the average diameter of NPs was around 165 nm (PDI: 0.32). SEM micrographs revealed that the NPs had a spherical morphology (Figure 2).

**Fig. 3:**
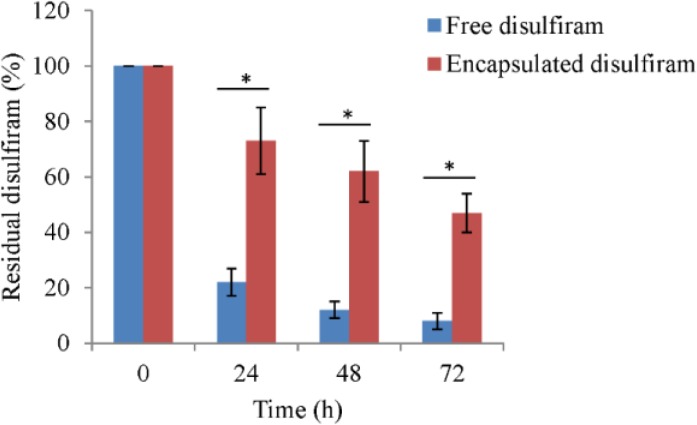
Disulfiram stability in its free or encapsulated form. The values are mean (n=3) ± SD. * p< 0.05

The results of disulfiramtability test showed that the free disulfiram is more degraded in solution (PBS containing 10% FBS) compare to encapsulated drug. After 24 h of experiment 22 ± 5% and 73 ± 12% of disulfiram remains in solution for disulfiram and encapsulated disulfiram, respectively. After 72 h, only 8 ± 3% of disulfiram remains for free disulfiram group, but in encapsulated disulfiram group 47 ± 7% of disulfiram remains in solution (Figure 3). 

**MTT Cell Proliferation Assay**

**Fig 4:**
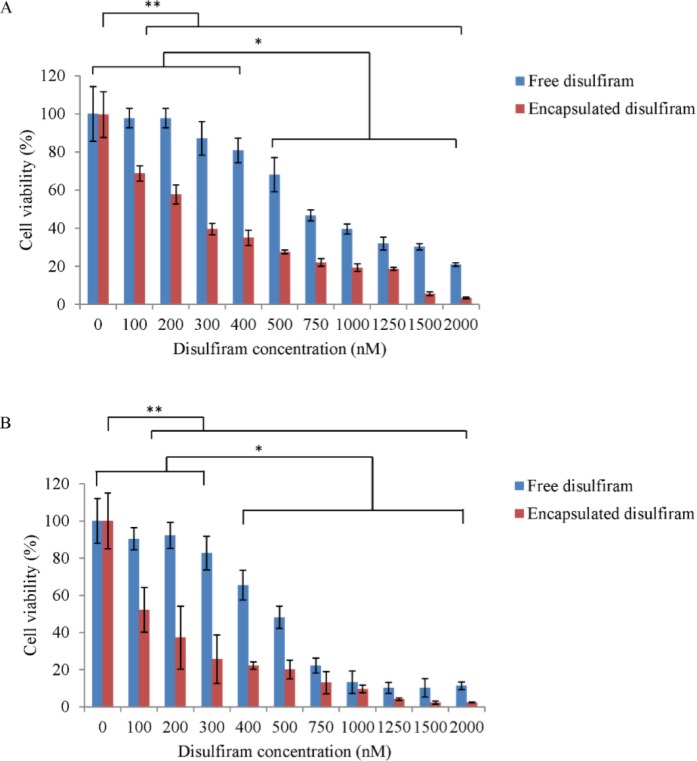
Cytotoxicity of disulfiram encapsulated PLGA NPs compared to free disulfiram on MCF7 cells after 24h (A) and 48h (B). The values are mean (n=3) ± SD. * p< 0.05 (free disulfiram); ** p< 0.05 (encapsulated disulfiram).

The in vitro cytotoxic effect of free disulfiram and disulfiram encapsulated PLGA NPs on MCF7 cells is presented in Figure 4. The result indicates that the drug formulated in the PLGA NPs had better advantaged in achieving higher cytotoxicity compared to the free drug. The IC_50_ dose of free disulfiram in 24h and 48h was around 750 nM and 500 nM, respectively. However, the IC_50 _dose of encapsulated disulfiram in 24 h and 48 h was around 300 nM and 200 nM, respectively. The un-encapsulated NPs had no cytotoxicity against MCF-7 cells. 

## Discussion

 Disulfiram has been approved by the Food and Drug administration for the treatment of alcoholism in 1951 by inhibition of aldehyde dehydrogenase.^21^^,^^22^The FDA-recommended average daily maintenance dose of disulfiram is < 250 mg.^22^The clinical trials for disulfiram show that this drug has an acceptable side-effect profile in FDA approved dose.^23^ The in vitro studies show that in toxic dose for cancer cell lines, disulfiram has no cytotoxicity effects against normal cell lines.^.^^14^^, ^^22^^, ^^24^  A growing body of studies revealed that disulfiram exerts a significant anticancer activity in vitro and in vivo.^22^^,^^24^ But it’s instability in gastric environment and rapid degradation in blood stream hampered its clinical usage for cancer therapy.^25^ Development of disulfiram formulation in a form of encapsulation in polymeric nanoparticles and its specific delivery into cancer cells can be alternative for its current oral use in cancer therapy. In this study, disulfiram was encapsulated into PLGA nanoparticles. The NPs were used for delivery of disulfiram into MCF7 breast cancer cell line. In vitro cell viability test indicated that disulfiram encapsulated NPs are far more cytotoxic than free disulfiram. This high cytotoxicity effect of disulfiram encapsulated NPs is not because of PLGA itself. PLGA is an FDA-approved biocompatible polymer and a trace amount of the PVA which was used as emulsifier in NPs preparation process could not be the cause of observed cytotoxicity. So, the higher cytotoxicity of encapsulated disulfiram could be due to more disulfiram delivery into breast cancer cells, its protection and appropriate release manner.

Moreover, it has been reported in the literature that PLGA NPs could have a higher cell uptake compared to the free drug.^20^^,^^26^ The results of this study are in accordance with Duan (2013),^24^Duan (2014),^27^ and Liu (2014)^14^in which they showed that encapsulated disulfiram have more stability and anti-cancer activity against cancer cells compared to the free drug. 

## CONCLUSION

 PLGA NPs can effectively be used for clinical application in order to delivery of disulfiram into tumor cells. This formulation can prevent rapid degradation of disulfiram in gastric environment and blood system and can provide a sustained release of this drug into tumor cells.
